# Peripheral Coding of Sex Pheromone Blends with Reverse Ratios in Two *Helicoverpa* Species

**DOI:** 10.1371/journal.pone.0070078

**Published:** 2013-07-23

**Authors:** Han Wu, Chao Hou, Ling-Qiao Huang, Fu-Shun Yan, Chen-Zhu Wang

**Affiliations:** State Key Laboratory of Integrated Management of Pest Insects and Rodents, Institute of Zoology, Chinese Academy of Sciences, Beijing, China; INRA-UPMC, France

## Abstract

The relative proportions of components in a pheromone blend play a major role in sexual recognition in moths. Two sympatric species, *Helicoverpa armigera* and *Helicoverpa assulta*, use (*Z*)-11-hexadecenal (Z11–16: Ald) and (*Z*)-9-hexadecenal (Z9–16: Ald) as essential sex pheromone components but in very different ratios, 97∶3 and 7∶93 respectively. Using wind tunnel tests, single sensillum recording and *in vivo* calcium imaging, we comparatively studied behavioral responses and physiological activities at the level of antennal sensilla and antennal lobe (AL) in males of the two species to blends of the two pheromone components in different ratios (100∶0, 97∶3, 50∶50, 7∶93, 0∶100). Z11–16: Ald and Z9–16: Ald were recognized by two populations of olfactory sensory neurons (OSNs) in different trichoid sensilla on antennae of both species. The ratios of OSNs responding to Z11–16:Ald and Z9–16:Ald OSNs were 100∶28.9 and 21.9∶100 in *H. armigera* and *H. assulta*, respectively. The Z11–16:Ald OSNs in *H. armigera* exhibited higher sensitivity and efficacy than those in *H. assulta,* while the Z9–16:Ald OSNs in *H. armigera* had the same sensitivity but lower efficacy than those in *H. assulta*. At the dosage of 10 µg, Z11–16: Ald and Z9–16: Ald evoked calcium activity in 8.5% and 3.0% of the AL surface in *H. armigera*, while 5.4% and 8.6% of AL in *H. assulta*, respectively. The calcium activities in the AL reflected the peripheral input signals of the binary pheromone mixtures and correlated with the behavioral output. These results demonstrate that the binary pheromone blends were precisely coded by the firing frequency of individual OSNs tuned to Z11–16: Ald or Z9–16: Ald, as well as their population sizes. Such information was then accurately reported to ALs of *H. armigera* and *H. assulta*, eventually producing different behaviors.

## Introduction

Male moths are attracted to females by conspecific sex pheromones, usually blends of two or more components released by females in defined ratios. In closely related moth species, pheromone blends often consist of the same compounds but in different ratios [1], [2]. Therefore male moths locate their mates based not only on the identity of pheromone components, but also on their ratios [2], [3]. Differences in blend ratios often underlie sexual isolation among sympatric species that may frequently encounter each other without mating.

How male moths discriminate sex pheromones released and successfully locate their mates is an active area of research [4]–[6]. Odorant molecules are first detected by receptors in the dendritic membrane of specialized olfactory sensory neurons (OSNs) housed in olfactory sensilla on the antenna, from which odor information is relayed to the antennal lobe (AL) [7], [8]. The OSNs are responsible for encoding the quality, quantity and temporal changes of the olfactory stimulus [9]. OSNs housed in trichoid sensilla of male moths usually detect sex pheromones, as reported in many Lepidopteran species [10]–[15]. In the AL, the OSNs expressing the same odor receptor converge onto a single glomerulus [16], [17]. An enlarged glomerular neuropil structure called the macroglomerular complex (MGC) is responsible for processing sex pheromones in the male AL [18]–[21]. Many studies have demonstrated the specificity of response of insect OSNs to individual pheromone components but it is still unclear how insects are able to determine specific ratios of these components based on peripheral coding.

The peripheral coding might be affected by the way the OSNs tuned to different components of the pheromone blend are distributed in functional types of trichoid sensilla. At present, two modes of distribution have been described. In the first mode, occurring in some families such as pyralid and saturniid moths, the OSNs tuned to different pheromone components are co-compartmentalized in the same sensillum. For example, both E and Z strains of *Ostrinia nubilalis* Hübner use (*Z*)-11- and (*E*)-11-tetradecenyl acetate (Z11- and E11–14: OAc) as their sex pheromones but in reverse ratios, 97∶3 and 1∶99, for the E and Z strains, respectively. [22]. Males distinguish between the two blends using OSNs co-compartmentalized in the same trichoid sensillum [23]. The major sex pheromone component of each strain elicits higher spike frequencies and larger spike amplitudes from corresponding OSNs than the minor component does from its corresponding OSNs [23], [24]. Moreover, the OSNs producing larger spike amplitudes have a larger dendritic diameter [25]. In the second distribution mode, reported in several large insect groups such as noctuid moths, the OSNs responding to individual pheromone components are housed in different trichoid sensilla. Single sensillum recordings have shown that in most heliothine moths, *Heliothis* and *Helicoverpa* species, divergent types of sensilla house OSNs that are tuned to different principal sex pheromone components [9]–[14], [26]–[28]. Typically, a larger fraction of OSNs responds to the major components of sex pheromones in heliothine moths [11], [13], [14], [29], [30], possibly to increase the sensitivity for pheromones. Baker et al. (2012) recently suggested that the functional reason for over-representation of OSNs for major pheromone components on moth antennae is to accommodate the greater dynamic flux rates encountered for the major pheromone component while maintaining overall response ratios [31]. In their conceptual paper, they find support to such hypothesis in the different dendrite sizes of OSNs in the co-compartmentalized OSN phenotype. However, an empirical study specifically relating ratio coding to neuronal populations is still lacking.

*Helicoverpa armigera* and *Helicoverpa assulta* are two sympatric, closely-related species based on morphological characters, genetic markers and pheromone production [32]–[34]. They both use (*Z*)-11-hexadecenal (Z11–16: Ald) and (*Z*)-9-hexadecenal (Z9–16: Ald) as principal sex pheromones but in different ratios [35]–[37]. Females of *H. armigera* produce 97% Z11–16: Ald and 3% Z9–16: Ald whereas *H. assulta* females produce 7% Z11–16: Ald and 93% Z9–16: Ald, ensuring premating-isolation of the two species. Wind tunnel and field trapping tests have demonstrated that the binary blends of Z11–16: Ald and Z9–16: Ald at their pheromone gland ratios are sufficient for eliciting male attraction behavior in the corresponding species [35]–[38]. This fact strongly suggests that the males of each species can recognize the ratio of the binary pheromone blend released by their own females. These two sympatric species therefore provide a good model for gaining insight into whether and how sex pheromone blend ratios are encoded at the peripheral level.

In order to understand how the OSNs encode a specific pheromone ratio and how the OSN-encoded information is processed in the ALs, in this paper we comparatively investigate pheromone reception on the antennae and in the ALs of *H. armigera* and *H. assulta*. First, we physiologically characterized the tuning properties of OSNs and monitored their relative abundance and distribution on the antenna of the two species. Second, we compared the responses of OSNs to binary blends at different ratios and investigated the processing of such information in the AL by calcium imaging. Third, we studied the behavioral responses of males to different binary blends in a wind tunnel.

## Results

### Dose-response Characteristics of OSNs Sensitive to Z11–16:Ald and Z9–16:Ald

**Figure 1:**
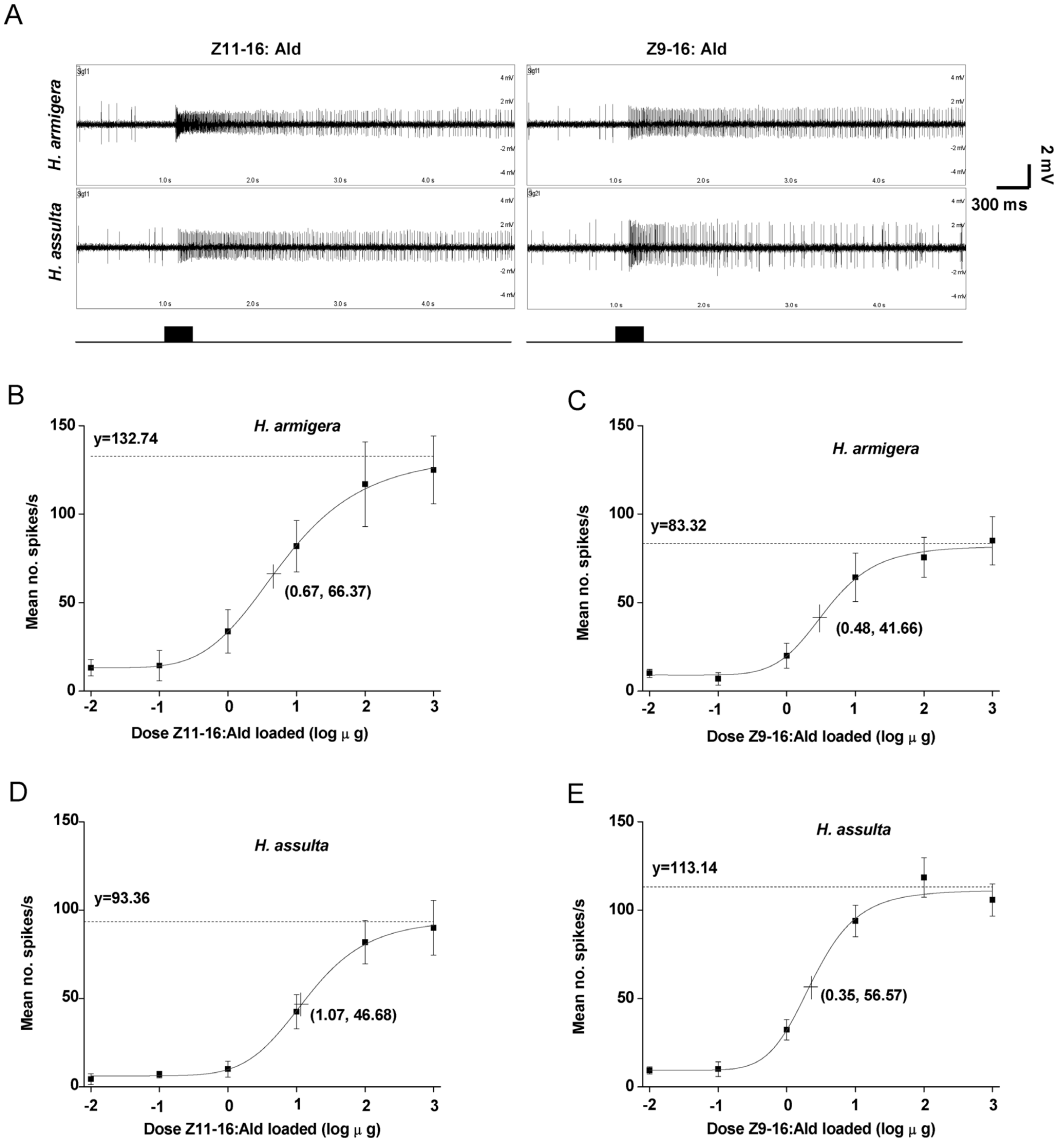
Dose–response curves of olfactory sensory neurons (OSNs) in two *Helicoverpa* species to Z11–16:Ald and Z9–16:Ald. **A**, typical response of OSNs responding to Z11–16:Ald and Z9–16:Ald in *H. armigera* and *H. assulta*. **B** and **C,** dose–response curve for Z11–16:Ald and Z9–16:Ald in *H. armigera*, respectively. **D** and **E,** dose–response curve of Z11–16:Ald and Z9–16:Ald in *H. assulta*, respectively. The fitted lines represent the following logistic regression equations: **B**, y = 132.743/(1+2.718exp(0.91–1.357x)), r^2^ = 0.99, n = 8; **C**, y = 83.32/(1+2.718exp(0.965–2.002x)), r^2^ = 0.98, n = 8; **D**, y = 93.364/(1+2.718exp(2.068–1.931x)), r^2^ = 0.99, n = 8; **E**, y = 113.144/(1+2.718exp(0.838–2.402x)), r^2^ = 0.98, n = 13. Numbers -2–3 on the X axis represent the following series of loaded dosages of Z11–16:Ald or Z9–16:Ald: 0.01, 0.1, 1, 10, 100 and 1000 µg. Spike frequency (spikes/s) was calculated by counting the actual number of spikes occurring during the first 200 ms of the response. Plotted values are mean ± SEM. The dotted line asymptotic to the curve indicates the maximal firing rate. The dosages of Z11–16:Ald and Z9–16:Ald inducing responses 50% of each maximal firing rate are indicated by a crossed symbol on the curve.

The OSNs sensitive to Z11–16:Ald were identified on the antennae of male *H. armigera* and *H. assulta* by single-sensillum recording (Figure 1A). In these two species, the dose-response characteristics of the OSNs were fitted to a logistic function, which revealed that *H. armigera* exhibited a higher maximal firing rate and higher sensitivity to Z11–16:Ald than *H. assulta* (Figure 1B, D). The 50% maximal firing rates of the Z11–16:Ald-sensitive OSNs in *H. armigera* and *H. assulta* were 66 and 47 spikes/s, respectively, and occurred at dosages of 5 and 12 µg, respectively (Figure 1B, D).

The OSNs sensitive to Z9–16:Ald in both *H. armigera* and *H. assulta* were also identified (Figure 1A), and their dose-response characteristics fitted to a logistic function (Figure 1C, E). The OSNs tuned to Z9–16:Ald basically had the same sensitivity in *H. armigera* and *H. assulta,* but the maximum firing rate was higher in *H. assulta* than in *H. armigera* (Figure 1C, E). The 50% maximal firing rates of those neurons in *H. armigera* and *H. assulta* were 42 and 57 spikes/s, respectively, at dosages of Z9–16:Ald of 3 and 2 µg, respectively (Figure 1C, E).

The OSNs sensitive to Z11–16:Ald and Z9–16:Ald displayed no cross-sensitivity to the two pheromone components although the two compounds are structural isomers (Figure S1). The OSNs tuned to Z11–16:Ald in *H. armigera* did not respond to Z9–16:Ald (Figure S1A, B), and the OSNs tuned to Z9–16:Ald in *H. assulta* did not respond to Z11–16:Ald (Figure S1A, C). The responses of the two OSN-types to the binary mixtures were only dependent on the dose of the corresponding components in the mixtures (Figure S1). Therefore, the binary mixtures were encoded along with the labeled-line principle.

### Abundance and Distribution of OSNs Sensitive to Z11–16:Ald and Z9–16:Ald in Antennae

Of the 413 trichoid sensilla investigated on 135 antennae of *H. armigera* males, 26.1% were classified as Z11–16:Ald type, and 7.6% as Z9–16:Ald type, while the remaining 66.3% did not respond to either of the two compounds. In *H. assulta*, we recorded from 371 trichoid sensilla on 103 male antennae and observed that 6.0% were sensitive to Z11–16:Ald, 27.2% were sensitive to Z9–16:Ald, and 66.9% showed no response to either. The ratios of the Z11–16:Ald-sensitive OSNs to the Z9–16:Ald-sensitive OSNs were 100∶28.9 in *H. armigera* and 21.9∶100 in *H. assulta*.

**Figure 2:**
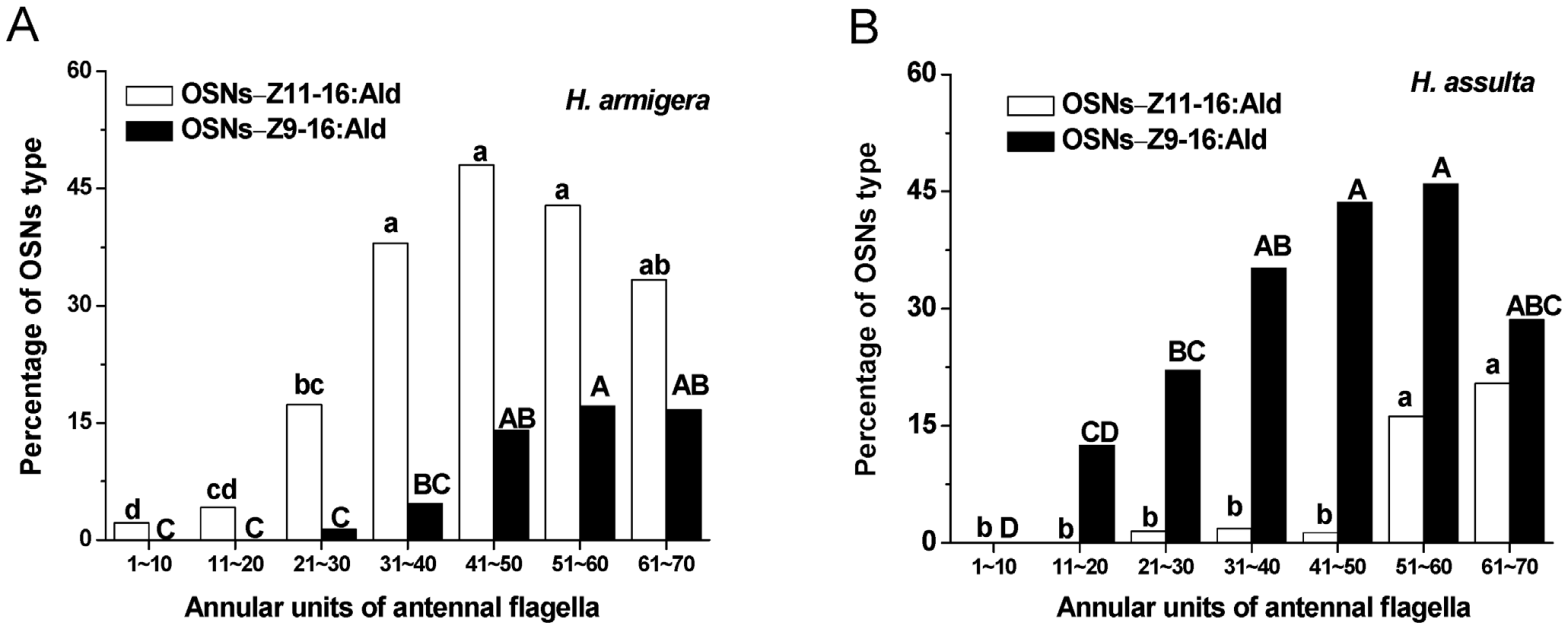
Percentages of OSNs sensitive to Z11–16:Ald and Z9–16:Ald in male antennae of two *Helicoverpa* species. The annuli are numbered 1–70 from the proximal to the distal end of the flagellum and each block of ten annuli was defined as one unit. Bars followed by the same letters are not significantly different from one another (*P*<0.05).

The distribution patterns of Z11–16:Ald-sensitive and Z9–16:Ald-sensitive OSNs along the antennal segments were different between *H. armigera* and *H. assulta* (Figure 2). Z11–16:Ald-sensitive OSNs were found in all annuli along the flagellum of male antenna in *H. armigera*; however, most of them were located between annuli 31 and 70, and the highest density was scored on annuli 41–50 (Figure 2A). In *H. assulta*, the Z11–16:Ald OSNs were mainly distributed in annuli 51–70 and were most dense in 61–70; none was found in annuli 1–20 (Figure 2B).

The Z9–16:Ald-sensitive OSNs were located in almost all of the annuli (11–70) along the flagellum of male antenna in *H. assulta*. They were mainly distributed in annuli 31–70, especially in annuli 51–60 (Figure 2B). In *H. armigera*, the Z9–16:Ald-sensitive OSNs were mainly distributed in annuli 41–70 and were most dense in 51–60; none was found in annuli 1–20 (Figure 2A).

### Relative Responses of OSNs to Binary Mixtures

**Figure 3:**
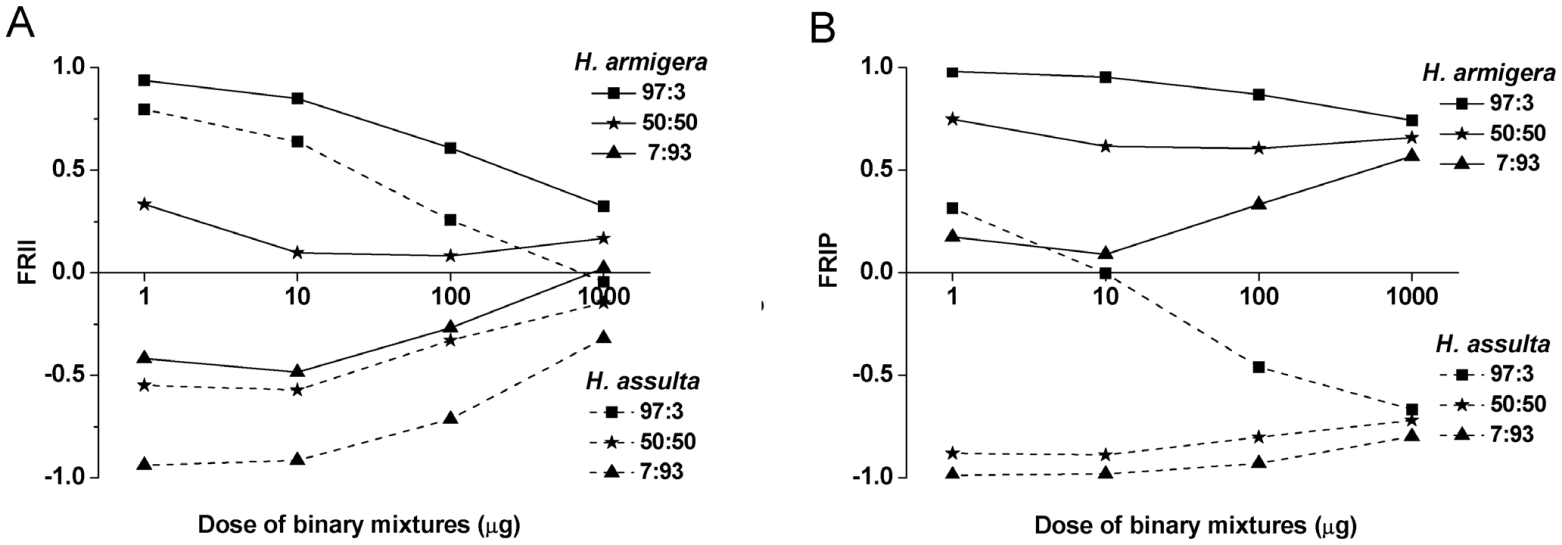
Relative response strengths of OSNs in two *Helicoverpa* species to binary pheromone mixtures. **A**, the firing rate index of individual OSNs (FRII); **B**, the firing rate index of OSN populations (FRIP). 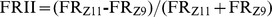 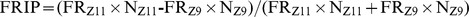 Here FR_Z11_ and FR_Z9_ are the firing rate of a single OSN tuning to Z11–16:Ald and Z9–16:Ald, respectively, to a given binary mixture at certain dosage; N_Z11_ and N_Z9_ are the relative percentages of the OSN populations tuning to Z11–16:Ald and Z9–16:Ald, respectively. Ratios of the two compounds are Z11–16: Ald to Z9–16: Ald.

We used the firing rate index of individual OSNs (FRII) and the firing rate index of OSN populations (FRIP) to compare relative response intensities of individual OSNs and whole OSN populations on the antenna to different binary mixtures (see Materials and Methods). In each species, the relative responses to 97∶3, 50∶50 and 7∶93 blends were distinctly different at both individual and population levels (Figure 3). The FRII and FRIP in *H. armigera* to the 97∶3 blend at different dosages correlated inversely with the same indices in *H. assulta* to the 7∶93 blend, but the FRIP remained more stable than the FRII at different dosages (Figure 3). We observed a convergent trend in FRII with increasing dosages (Figure 3A), but a clear separation in FRIP between the two species (Figure 3B).

### Spatial Representation and Dose-response Curve of Z11–16:Ald and Z9–16:Ald in the AL

**Figure 4:**
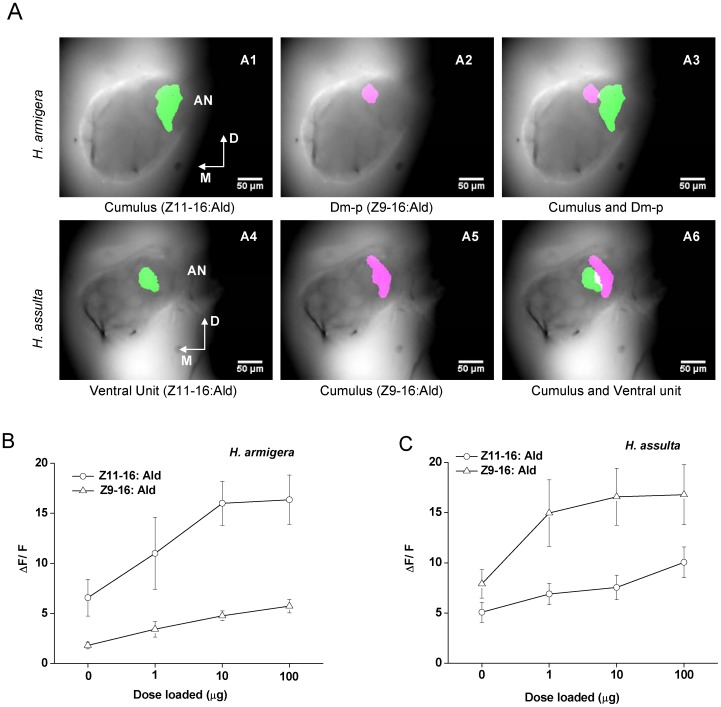
Spatial representation and dose-responses of Z11–16: Ald and Z9–16: Ald in the antennal lobes (AL). Green was chosen for activated area of Z11–16: Ald and pink for Z9–16: Ald. **A**, A1-A3 show the spatial representation and relative position of Z11–16: Ald and Z9–16: Ald in the AL of *H. armigera*, A4-A6 show those of *H. assulta*. D,dorsal; M, medial; Dm-p, posterior dorsomedial unit. **B**, Dose-response curve of Z11–16: Ald and Z9–16: Ald in the AL of *H. armigera*. **C**, Dose-response curve of Z11–16: Ald and Z9–16: Ald in the AL of *H. assulta*. Values are mean ± SEM (*H. armigera*, n = 5; *H. assulta*; n = 7).

Based on the AL atlas of *H. armigera* and *H. assulta* [39], [40], we were able to identify the activated MGC units based on their position and outline. The activity patterns (frontal view) shown in Figure 4 were evoked by stimulation with 10 µg of Z11–16: Ald and Z9–16: Ald. The cumulus of the MGC of *H. armigera* was activated by Z11–16: Ald, as shown by the pseudo-colored activity region close to the entrance of antennal nerve into the AL (Figure 4 A1). The posterior dorsomedial glomerulus (Dm-p) was activated by Z9–16: Ald in the same AL (Figure 4 A2). In *H. assulta*, Z11–16: Ald activated a smaller ventral subunit of the MGC (Figure 4 A4), while Z9–16: Ald activated the cumulus (Figure 4 A5). Such reversed topology of activation patterns of these two compounds in the AL of the two species was consistent among individuals. Interestingly, while the areas activated by Z11–16: Ald and Z9–16: Ald were spatially separated in *H. armigera*, some overlap was observed in the activity pattern evoked by these two compounds in *H. assulta*.

The total areas of the AL surface in *H. armigera* and *H. assulta* in imaging were 35631±1141 µm^2^ and 33440±887 µm^2^, respectively, and were not statistically different between the two species (Levene’s test, n = 12, F = 0.835, *P* = 0.144). In *H. armigera*, the area activated by Z11–16: Ald was 8.5% of the AL and that activated by Z9–16: Ald only 3.0%, while in *H. assulta* the values were 5.4% and 8.6%, respectively.

The dose-response dynamics of the two compounds were markedly different between the two species (Figure 4B, C). The intensity of responses to Z11–16: Ald was stronger than that to Z9–16: Ald at any tested dosage in *H. armigera*, but the reverse was observed in *H. assulta*. The responses to the major sex pheromone component in both species showed clear saturation at 10 µg, but there was no saturation up to 100 µg for the minor ones (Figure 4B, C). The spatial pattern upon activation by single components at different dosages remained unchanged (data not shown).

### Glomerular Activities in Antennal Lobes Induced by Binary Pheromone Mixtures

**Figure 5:**
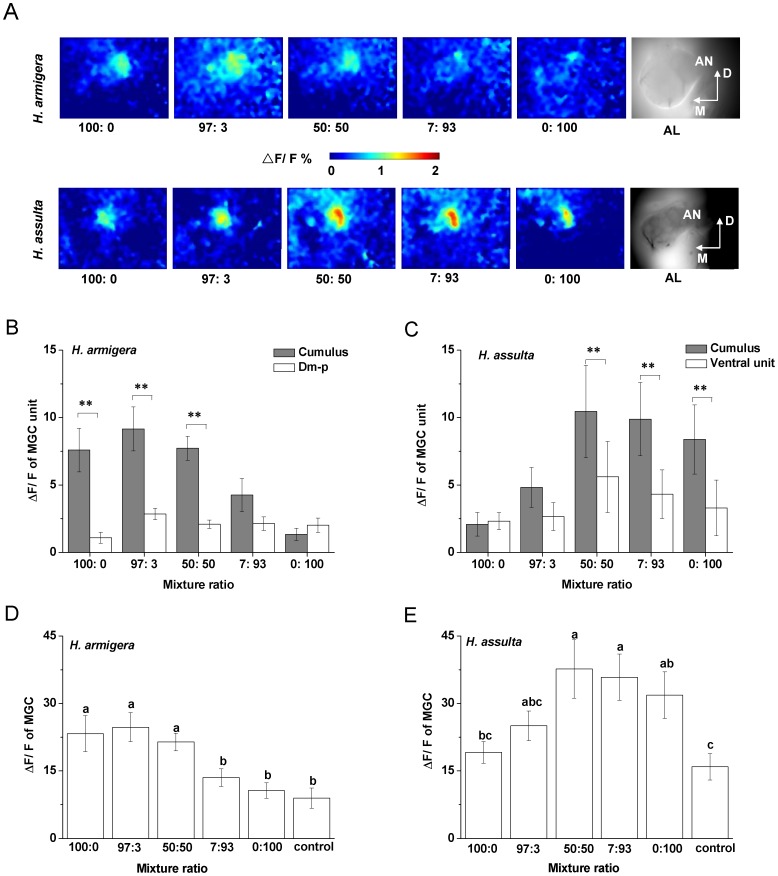
Glomerular activities in antennal lobes induced by binary pheromone mixtures at different ratios. MGC, macroglomerular complex. Ratios of the two compounds are Z11–16: Ald to Z9–16: Ald. 10 µg was used as stimulus dose. **A**, peak activities to different binary mixtures in false-color scale in *H. armigera* and *H. assulta*. For each species, all the pictures were taken on the same optical plane and had the same size (320×240 pixels); the last picture of the row shows the AL structure. D, dorsal; M, medial. **B** and **C**, net responses of MGC units to binary mixtures at different ratios in *H. armigera* and *H. assulta*. Values are mean ± SEM. (Paired t-test, ** *P*<0.01, *H. armigera*, n = 6; *H. assulta*, n = 6). **D** and **E**, response intensity of whole MGC to binary mixtures at different ratios in *H. armigera* and *H. assulta*, respectively. Values are mean ± SEM. The same letters above bars are not significantly different from one another (ANOVA, *P*<0.05, *H. armigera*, n = 6; *H. assulta*, n = 6).

The MGC units activated by Z11–16: Ald and Z9–16: Ald were spatially separated in the AL of *H. armigera* (Figure 5A). However, in *H. assulta*, the cumulus of the MGC covered the ventral unit which was innervated by OSNs responsive to Z11–16: Ald (Figure 5A). In the two species, when stimulating the preparations with mixtures of the two components in different ratios (97∶3; 50∶50; 7∶93), the two MGC units were activated in a combinatorial manner although the stronger response was usually found in the cumulus. Moreover, 97∶3 and 50∶50 blends produced stronger responses in *H. armigera*, while 7∶93 and 50∶50 blends produced stronger responses in *H. assulta* (Figure 5A).

The response intensities of MGC units and of the whole MGC to different mixtures at a dosage of 10 µg were in agreement with the ratio discrimination by OSN populations (Figure 3B). In *H. armigera*, mixtures 100∶0, 97∶3 and 50∶50 induced significantly stronger responses in the cumulus than in the Dm-p (Figure 5B), and also induced stronger responses in the whole MGC relative to other mixtures (Figure 5D). In *H. assulta*, mixtures 50∶50, 7∶93 and 0∶100 evoked stronger responses in the cumulus than in the ventral unit (Figure 5C), and also induced stronger responses in the whole MGC with respect to other mixtures (Figure 5E). Therefore, in both species, a stronger response in the cumulus or in the MGC was associated with a larger proportion of the major pheromone component in the blends (Figure 5).

### Behavioral Response of Males to Binary Mixtures

**Figure 6:**
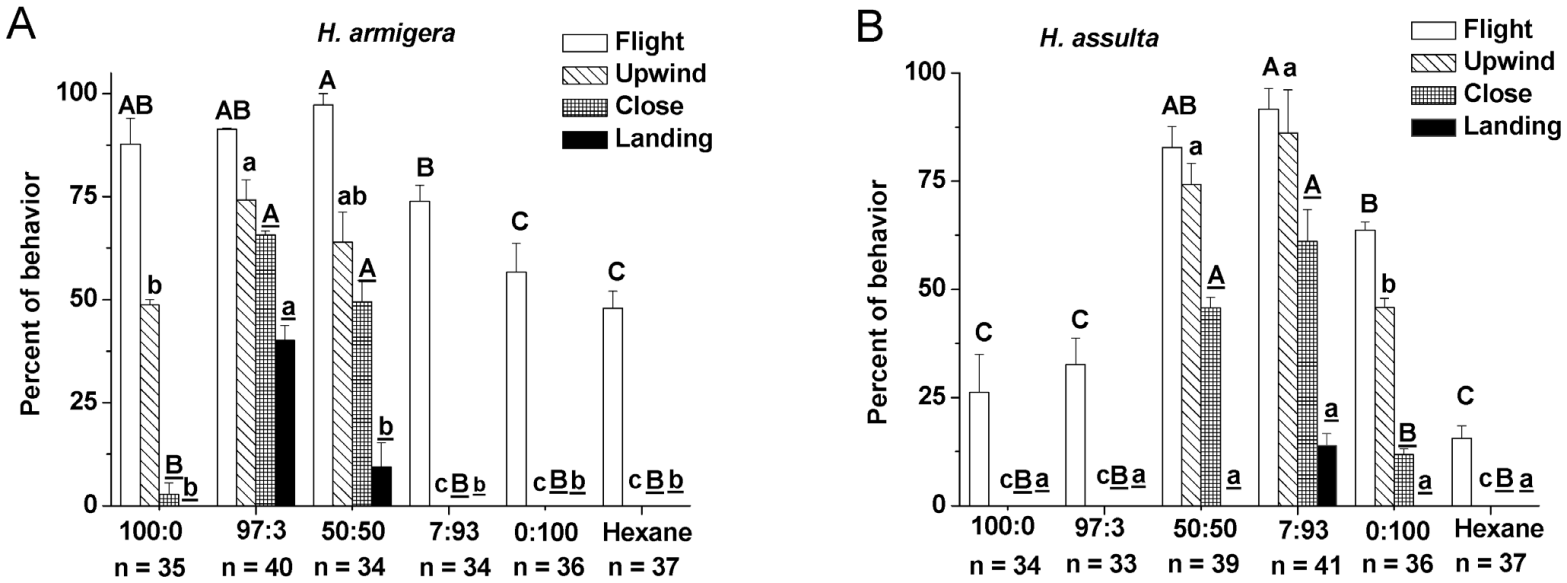
Behavioral responses of males to binary mixtures at different ratios. **A, ***H. armigera*; B, *H. assulta*.**** Ratios of the two compounds are Z11–16: Ald to Z9–16: Ald. Values are mean ± SEM. Symbols in the same behavioral category having no same letters are significantly different (Chi-square test, *P*<0.05).

The major component (Z11–16: Ald for *H. armigera* and Z9–16: Ald for *H. assulta*) alone could elicit upwind flight in both species, but addition of the secondary component significantly increased the frequency of the upwind flight and close contact, indicating that the secondary component was required for close range orientation of males (Figure 6). In the case of *H. armigera*, males failed to show upwind flight and subsequent behaviors in response to the 7∶93 and 0∶100 blends, suggesting that the males did not recognize these stimuli as sex pheromones (Figure 6A). The same phenomenon was also found with *H. assulta* males when stimulated with mixtures 100∶0 and 97∶3 (Figure 6B). Applying the optimal blend of 97∶3 for *H. armigera*, 40% of males contacted the odor source (Figure 6A). With *H. assulta*, the optimal blend of 7∶93 produced a weaker effect, with only 14% of the moths contacted the odor source, a result not significantly different from those obtained with the other blends (Figure 6B).

## Discussion

The main barrier of prezygotic isolation between the sympatric sibling species *H. armigera* and *H. assulta* is their sex pheromones [41]. The differences in the ratio of sex pheromone components released by females and in behavioral responses of males to these pheromones result in the absence of mutual attraction between sexes of different species [38], [42]. In this study, we found that the sex pheromone blends of the two species were precisely coded by the firing frequency of individual OSNs tuned to Z11–16: Ald or Z9–16: Ald, as well as by the proportions of OSNs tuned to each of the two components; the response intensities from the peripheral to AL were well correlated to the behavioral preference for ratios of the two components.

### Firing Frequency of Individual OSNs and the Size of the Responding Receptor Populations Encode Ratios of the Sex Pheromone Blends

Pheromone component ratio detection is essentially based on comparing the relative strengths of sensory input generated by components in the mixture [43]. The frequency of discharge of an OSN underlies the neural code for the stimulus strength, and this property of sensory neurons is called the frequency code [44]. The firing rate of OSNs responding to pheromone components is important for detecting changes of components concentrations [9], [26]. To enable recognition of their sex pheromones, the heliothine moths possess highly selective OSNs within the sensilla trichodea of male antennae [9]–[11]. Our study confirms that two separate groups of OSNs are present in different sensilla on the antennae of *H. armigera* and *H. assulta.* They are tuned to Z11–16:Ald and Z9–16:Ald respectively, with no cross responses to each other (Figure 1A, Figure S1). The same group of OSNs between the two species showed differences in sensitivity and efficacy, and the OSNs tuned to the major pheromone components had higher efficacy than those tuned the minor components in both species (Figure 1).

In addition to the discharge frequency of individual OSNs, the size of the population of responding OSNs also provides a neural code, called a population code [44]. The ratios between Z11–16:Ald-sensitive OSNs and Z9–16:Ald-sensitive OSNs in *H. armigera* and *H. assulta* were 100∶28.9 and 21.9∶100, respectively, while the ratios between Z11–16:Ald and Z9–16:Ald in their sex pheromone glands are 100∶2.1 and 5.8∶100, respectively [45]. Similar results have been reported in *H. zea*, with estimates of the ratio between the sex pheromone components Z11–16:Ald and Z9–16:Ald ranging from 100∶1.6 to100∶2.4 [46]–[48], and the ratio between the corresponding two types of OSNs in the male antenna being 100∶26.8 [14]. It is clear that the largest numbers of OSNs are always tuned to the most abundant pheromone component in sex pheromone blends [11], [12], [29], [30]. Therefore, population codes also play an important role in coding sex pheromone ratios.

The functional reason for over-representation of OSNs responding to major pheromone components on moth antennae deserves discussion. Baker et al. (2012) proposed that such an arrangement is to accommodate the greater dynamic flux rates encountered for the major pheromone component while maintaining overall response ratios unaffected [31]. Thus functional adaptations towards high flux rates of molecules may make the sensilla/OSNs for major pheromone components less sensitive than those for the minor pheromone components. Such over-representation would be a way to compensate for lower sensitivity. However, this is not supported by our results, nor in other studies. In *H. armigera*, the Z11–16:Ald OSNs and Z9–16:Ald OSNs had similar sensitivity, while in *H. assulta*, the Z9–16:Ald OSNs had higher sensitivity than the Z11–16:Ald OSNs (Figure 1). In *H. zea*, the major sex pheromone component Z11–16:Ald also evoked a higher spiking frequency than Z9–16:Ald at the same dosage [14]. In E- and Z-strains of *O. nubilalis*, the major pheromone isomer of each strain elicited larger spike amplitudes than the minor one [23], [24] and the large-spiking neurons were slightly more sensitive than the small-spiking neurons [23]. Hansson et al. (1994) also found that ORNs with greater diameter produced spikes with larger spike amplitude, and then suggested that a larger number of ion channels could be available to detect the major pheromone component, thereby increasing neuronal sensitivity [25]. We suggest that the relative abundance of OSNs of each type is an important factor improving the accuracy of ratio detection. Over-representation of some OSN types on the antenna, therefore, is just another extension of the need for more olfactory receptor proteins predicted by kinetic models for accommodating greater flux rates [49].

### Calcium Imaging Results Correlate with Peripheral Input Signals and Behavioral Observations

The relative firing frequencies of OSNs accurately transmit the complete blend information to the AL [31]. Glomeruli are structural and functional units of AL, and their number and spatial arrangement is reproducible in congeneric species [50]. The OSNs expressing the same odor receptor converge onto one glomerulus, and thus the across-glomerular ratios of activity to different odorant blends in the AL could reflect the peripheral input signals. In this study, calcium green 2-AM was used in optical recording because its signal mainly presents the input information from OSNs [51]–[53]. Based on morphological atlases of AL in *H. armigera* and *H. assulta* [39], [40], calcium imaging results reveal that the OSNs tuned to Z9–16: Ald innervate the cumulus of MGC in *H. assulta* (Figure 4 A5), and OSNs responding to Z11–16: Ald arborize in a ventral unit (Figure 4 A4). This finding is in agreement with the former electrophysiological work combined with neuronal staining [12], [54]. On the contrary, in *H. armigera*, Z11–16: Ald activates the cumulus of the MGC whereas Z9–16: Ald activates only a smaller subunit, the Dm-p (Figure 4 A1, A2).

In these two species, the major pheromone-component-activated cumulus was characterized by a larger area. The glomerular zones activated by the major component were 2.81 and 1.58 times the size of those activated by the minor component in *H. armigera* and *H. assulta*, respectively. Dose-response curves of Z11–16: Ald and Z9–16: Ald in optical recordings show that the major component induces a stronger response in each of the two species at the same dosage (Figure 5). These findings prove the convergence of the same type OSNs in specified glomeruli. In other Lepidopteran species such as *H. virescens* and *H. zea*, as well as the Z and E strains of *O. nubilalis*, MGCs present similar structures, with the larger cumulus also dedicated to processing the major sex pheromone component [29], [30], [55], [56]. In *Drosophila* a strong correlation was found between the abundance of different types of OSNs and the volumes of corresponding glomeruli [57]. These results suggest that the relatively higher abundance of OSNs results in a large volume of its target glomerulus [58].

Insects discriminate different odor blends through combinatorial activation patterns in AL [59], [60]. The response patterns to the binary blends with different ratios indicate that component information is preserved, but the major component is salient in the mixture representation in both species (Figure 5), which is in agreement with previous studies in other systems [61], [62]. The AL activities evoked by the binary blends in the two species mirrored well the relative response strengths of antennal OSN populations. The responses in MGC units to the pheromone mixtures at the dosage of 10 µg (Figure 5B,C) were in agreement with the firing rate indices of whole OSN populations on the antenna at the same dosage (Figure 3B). It seems that these mixture responses are clustered by their composition, especially by the major component, indicating that the mixture representation could be predicted by their components and follows elementary processing in the AL [61]–[63].

Imaging results obtained with mixtures was also consistent with behavior of the two species in the wind tunnel. The mixtures of 100∶0, 97∶3 and 50∶50 for *H. armigera* as well as 50∶50, 7∶93, 0∶100 for *H. assulta* which evoked the strongest responses in the whole MGC (Figure 5D, E) also induced male upwind flight behavior in the two species (Figure 6). However, these mixtures had different effects on close-range orientation to the pheromone lure (Figure 6), suggesting that mixture-unique properties would further be resolved in higher brain centers [64]. Many studies have demonstrated that differences among mixtures are enhanced after antennal lobe processing [51], [65], [66].

**Figure 7:**
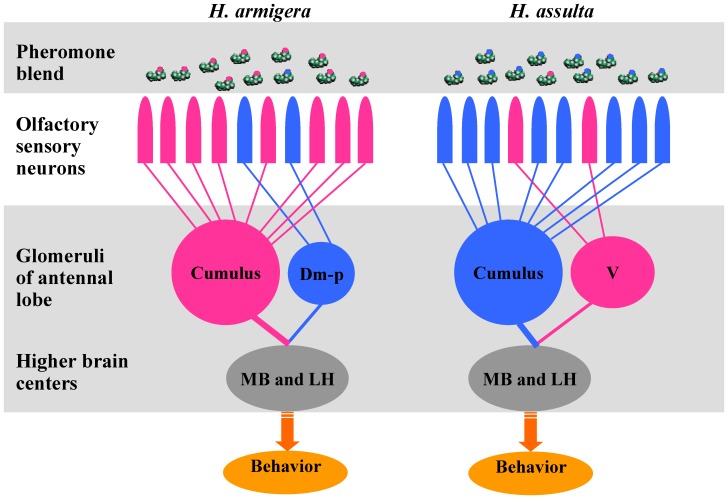
Olfactory processing to the binary pheromone mixtures with reversed ratios in two *Helicoverpa* species. Dm-p, posterior dorsomedial unit; V: ventral unit; MB: mushroom body; LH; lateral horn.

In summary, peripheral coding of the sex pheromone blends in both *H. armigera* and *H. assulta* can be attributed to two populations of highly specific OSNs in different antennal sensilla that detect binary blends of Z11–16:Ald and Z9–16:Ald with reversed ratios. The two groups of OSNs can be thought of as labeled lines, each sending unique information based on their firing frequency and population size, and continuously and accurately signaling to the antennal lobe the relative concentrations of Z11–16:Ald and Z9–16:Ald in the air. Such a high-fidelity signal transmission by the OSNs contributes to the selectivity and sensitivity of the behavioral responses in each species (Figure 7). The central nervous system detects mixture ratios at the level of the glomeruli or at higher olfactory centers (e.g., in the mushroom bodies and lateral horns) [6], [63], [64], and accordingly determines whether behavior is or is not released. Differences in the sex pheromone communication system underlie behavioral isolation between the two sympatric species. In future research, we will focus on the output information of AL and will explore how these mixtures at different ratios get subdivided at the level of higher brain areas.

## Materials and Methods

### Insects

*H. armigera* and *H. assulta* were collected in the crop fields from Zhengzhou, Henan Province, China. All collections were undertaken by State Key Laboratory of Integrated Management of Pest Insects and Rodents, Institute of Zoology, Chinese Academy of Sciences. For the two species are agricultural pests in China, no any specific permission was required to collect any of these samples. Successive generations were separately maintained in the laboratory under a 16L:8D photoperiod at 26±1°C and relative humidity 55–65%. Larvae were maintained on an artificial diet with wheat germ as the main ingredient [67]. Pupae of different sex were placed in separate cages. Emerged moths were collected daily, placed individually in cages and fed with 10% honey-water. Adult male moths used for electrophysiological studies were 2–6 days old.

### Sex Pheromone Components

The sex pheromone compounds Z11–16:Ald and Z9–16:Ald were purchased from Shin-Etsu Company (Tokyo, Japan). The purity upon purchase was 95% and further purification to 99% was carried out on a silica gel column. The compounds were verified using a capillary column gas-chromatograph (BP-20, i.d. 0.22 mm, length 25 m). Serial dilutions of these sex pheromone components were kept in redistilled HPLC-grade hexane or paraffin oil (Analytical grade, Fluka). The solutions were stored in 2 ml glass vials (Agilent Technologies, Santa Clara CA, USA) at −20°C.

### Sensitivity of Specialized OSNs to Pheromone Components and Mixtures

To characterize the sensitivity of the specialized OSNs to Z11–16:Ald and Z9–16:Ald in each insect species, we used the cut-sensillum technique to make single sensillum recordings (SSR) from the OSNs within an individual antennal sensillum [68]. OSNs were categorized as sensitive to Z11–16:Ald or sensitive to Z9–16:Ald according to their responses to a dose of 10 µg. For each compound, 10 µl of the 1 µg/µl solution was placed on a filter paper strip (0.7 cm×2.5 cm) held in a Pasteur pipette (15 cm long), hereafter referred to as the odor cartridge. Stimulus compounds were applied in random order at 30 s intervals. To generate dose-response curves, stimulus dilutions were prepared of 0.001, 0.01, 0.1, 1, 10 and 100 µg/µl and 10 µl of a dilution was applied at the filter paper as stimulus source. The sequence of stimulus delivery was from low to high concentration, at intervals >30 s. The concentrations in each solution were confirmed by capillary gas chromatography-mass spectrometry. The blank, 10 µl of redistilled HPLC-grade hexane loaded onto the odor cartridge, was applied every three stimulations. The blank response was subtracted from the adjacent stimulation response. For each treatment, at least eight recordings were obtained from different individuals. The dose-response mathematic formula of each type of OSN in the two species was derived using a logistic regression model, and the sensitivity of the tested neuron was determined as the dose that induced 50% of the maximal firing rate in the tested sensilla.

To test possible cross-sensitivity of OSNs to the two pheromone components, we investigated the firing frequencies of the two types of OSNs to the mixtures of Z11–16:Ald and Z9–16:Ald at different ratios (100∶0, 97∶3, 50∶50, 7∶93, 0∶100). For each mixture 10 µl of a 1 µg/µl solution was used as odor source, so the tested dosage was 10 µg. Paraffin oil was used as the control.

### Abundance and Distribution of OSNs Responding to Pheromone Components

The annuli were numbered 1–70 from the proximal to the distal end of the flagellum and single sensillum recordings were made from sensilla trichodea that were randomly selected on each annulus. Data were collected from 413 sensilla on 135 antennae of *H. armigera* males and from 371 sensilla on 103 antennae of *H. assulta* males. No recordings were made beyond the 70^th^ annulus because of technical difficulties. The annuli were grouped into seven sections from the antennal base to the tip, with each group of ten annuli considered to be an analytic unit. The relative ratios of the OSN populations tuned to Z11–16:Ald and Z9–16:Ald in male antennae of both species were measured.

### Relative Response Strengths of OSNs to Binary Mixture at Different Ratios

Based on the single sensillum recording, we identified the two groups of OSNs, and each of them selectively responded to one of the two sex pheromone components in the two species [9], [12], [13]. We assumed that pheromone molecule-receptor encounter is a random process, therefore Poisson statistics are adequate [69]. The summation of individual Poisson processes is still a Poisson process. Based on the above logistic equation for the dose-response curve and the relative abundance of OSNs of each type, we used two indices of firing rate to express the relative response strengths of two types of OSNs to binary mixture at different ratios: (1) the firing rate index of individual OSNs (FRII); (2) the firing rate index of OSN populations (FRIP). They are calculated as:
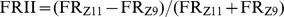


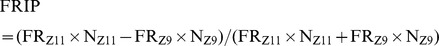



Here FR_Z11_ and FR_Z9_ are the firing rates of a single OSN tuning to Z11–16:Ald and FRZ9–16:Ald to a given binary mixture of the two compounds at certain dosage, respectively. They were calculated according to the logistic dose-response relationship obtained above (Figure 3). N_Z11_ and N_Z9_ are the relative percentages of the OSN populations tuned to Z11–16:Ald and Z9–16:Ald, respectively.

Since Z11–16:Ald and Z9–16:Ald at the dosages of 0.01 µg and 0.1 µg induced weak or no responses in the two species (Figure 1), we only calculated the FRII and FRIP of OSNs responding to the binary mixtures of 97∶3, 50∶50; 7∶93 at the dosages of 1, 10, 100, 1000 µg.

### Glomerular Activities in Antennal Lobes Induced by Single Pheromone Components and Binary Mixtures

To compare the spatial representation of Z11–16: Ald and Z9–16: Ald in the antennal lobe of these two *Helicoverpa* species, we checked the responses to single compounds (Z11–16: Ald and Z9–16: Ald) and their binary mixture at different ratios (97∶3, 50∶50; 7∶93) in the AL by calcium imaging. The used dosage was 10 µg, which was based on the dose-response curves in the single sensillum recordings. The activated MGC units were identified based on their position and outline described in the studies of the AL atlas of *H. armigera* and *H. assulta* [39], [40]. The three units of MGC of *H. armigera* were the cumulus, the posterior dorsomedial glomerulus (Dm-p), and the anterior dorsomedial glomerulus [40]. The three units of the MGC of *H. assulta* were the cumulus, a smaller ventral unit, and a dorsomedial unit [39].

In the single compound representation, we first determined the activated area in false color-coded images with peak activity at the response dosage 10 µg, and then recognized each MGC unit in the construction of AL. The activated area was identified and calculated according to their position and outline by ImageJ software (NIH, USA) (Figure S2). In previous optical imaging studies [51], [61], [70], the same size of responsive areas was chosen to compare the response amplitude to different odors, which represented the mean response of each pixel in the given glomerulus. In this study, sizes of the activated area were taken into account because the MGC units responsive to Z9–16: Ald and Z11–16: Ald made a large difference in the volume [39], [40]. The overall responses of the MGC units to Z9–16: Ald and Z11–16: Ald were calculated as the mean response amplitude of each pixel multiplied by the activated area.

In the representation of the mixtures, the overall responses of two activated MGC units and the total MGC were determined. The response of each activated MGC unit was calculated within an identified area determined in the single compound representation above, and the corresponding control was subtracted from the whole response in each unit. The response of the total MGC, was calculated within a large area (80×60 pixels) which was big enough to cover the MGC. For each sample, the consistent position and size was used to calculate responsive intensity among different treatments. More than five replicates were conducted.

### Behavioral Responses of the Males to Binary Mixtures

Wind tunnel experiments were done to compare the behavioral differences between *H. armigera* and *H. assulta* to a series of binary sex pheromone blends (Z11–16:Ald and Z9–16:Ald) with ratios of 100∶0, 97∶3, 50∶50, 7∶93, 0∶100. Hexane was used as the control. The 3-day old virgin males were tested during their scotophase (4–6 hr) in a plexiglas wind tunnel. The wind tunnel was 2.5×1×1 m (L×W×H). The conditions in the experiment were as follows: 22–25°C, 40–60% relative humidity and 0.3 Lux of red light. Wind speed was about 30 cm/s. Before behavioral recording, the males were moved to wind tunnel room to acclimate to the conditions for at least 40 minutes. Rubber septa were loaded with 1 mg (10 µl of 100 µg/µl solution) binary mixture of Z11–16: Ald and Z9–16: Ald at different ratios (100∶0, 97∶3, 50∶50, 7∶93, 0∶100). The loaded rubber septum was tied by a hook as pheromone source, at 30 cm from the upwind end and 40 cm above the floor. Individual males were put into cylindrical mesh cages (10 cm long and 5cm in diameter) and released downwind 2 m from the pheromone source and 30 cm above the floor. The behavioral responses of males were classified according to the following typical series: (1) Flight: male moths took off from release cage; (2) Upwind: male moths flew at the height of lure and showed characteristic zig-zag flight behavior towards pheromone source within 70 cm; (3) Close: male moths continued upwind behavior and flew within 10 cm of pheromone source; (4) Landing: male moths contacted the lure. Every male moth was recorded for 5 minutes. In one treatment, 9–15 male moths were tested on any given day. The order of treatments in one replicate was randomized. At least three replicates were run.

### Single Sensillum Recording

The antenna was cut off from the scape, the tip of a glass micropipette filled with hemolymph saline [71] was pushed into its open end, and the tip of the flagellum was immobilized with a solution of polyvinylpyrrolidone (PVP, 1 g/ml in water). The tip of one of the sensilla trichodea was then cut off using special fine forceps with sharp-edged tips, and the cut end was immediately brought into contact with another glass micropipette filled with receptor lymph saline [71]. To prevent leakage of receptor lymph or electrolyte, and to avoid drying out of the hair or electrode, the tip of the second micropipette was filled with a PVP solution (0.1 g/ml in receptor lymph saline), and the outside was sealed with Vaseline™. The electrical activities of the receptor cells in the sensilla were recorded through an Ag-AgCl electrode placed in the saline-filled micropipette.

The signals from the recording electrode were transmitted to a programmable signal-recording and output controller (IDAC-4, Syntech, Hilversum, The Netherlands). Autospike version 3.4 software (Syntech) was used for both recording and data analysis. A continuous stream of purified and humidified air was directed over the antenna (12.5 ml/s) from the outlet of a steel tube (i.d. 6 mm, length 15 cm), positioned 2 cm from the antenna. Test odors were injected into the air stream using a stimulus flow-controller (CS-55, Syntech), which generated 300 ms air pulses through the odor cartridge at a flow rate of 10 ml/s.

Action potential frequencies (spikes/s) were calculated by counting the number of spikes occurring during the first 200 ms of the response. For both non-responding and poorly responding OSNs, spikes were counted during the 200 ms stimulation period.

### Calcium Imaging

Calcium imaging was conducted as described previously [53], [61], [70]. In brief, the moths were restrained in plastic tubes and fixed with dental wax. Then the tube was fixed into a custom-made chamber and the antenna was fastened in the ideal position for odor stimulation. Scales were moved, a window was made in the head between two compound eyes, and then mouthparts, glands, muscles and tracheae were removed to expose the brain. During the preparation, the brain was kept in Ringer solution [20]. A calcium-sensitive dye CaGR-2-AM (Molecular Probes, Eugene, OR, USA) was used to stain the brain. The dye was firstly dissolved in 20% Pluronic-127 in dimethyl sulfoxide and then diluted by Ringer solution to a final concentration of ∼30 µmol/L. The animal was then placed in the dark for 1 hour at 12°C. After staining, the brain was thoroughly rinsed with Ringer solution several times and ready for imaging.

Imaging data were collected by using a Till-Photonics imaging system (Till Photonics, Germany). Monochromatic light was 475 nm, dichroic: 500 nm, and emission LP, 515 nm. A sequence of 40 frames was acquired with a sampling rate of 4 Hz and exposure time was 200 ms. All the measurements were recorded using an upright microscope (Olympus BX51WI, Tokyo, Japan) with a 20× (NA 0.95, Olympus) water immersion objective. Stimulation was set at frame 12 and lasted for 500 ms. The final size of image presentation was 320×240 pixels by binning 2×2 on chip. The stimulus delivering set-up was the same as the single sensillum recording.

Raw data were firstly filtered using spatial and time median filters with a radius of 2 pixels to remove “salt and pepper” noise [50]. Background fluorescence (F) was defined as the mean fluorescence of frames 2–11, which was just before onset of stimulation. For the false color images, the relative calcium change of each frame was calculated as relative changes in fluorescence (ΔF/F). In time traces, Fn/F (n = 1–40) was expressed for the ratio of each frame against background, and then followed by a smoothing treatment (smooth, 30). ΔF/F of each frame was defined as the difference between before and after smoothing, i.e. ΔF/F = Fn/F - Fn/F (smooth, 30). The resulting image was subsequently false-color coded. The smooth treatment can reduce noise but not remove pertinent signals. For quantitative analysis of the response intensity, the mean of three sequential frames at the signal’s maximum was taken as the amplitude of odor-induced responses.

### Data Analyses

The dose-response curves of ORN electrophysiological activity in *H. armigera* and *H. assulta* were analyzed by nonlinear regression (logistic model). A weighted average method was used for the analysis of the ratios of different functional types of sensilla along the whole antenna. One-way ANOVA analysis was used to compare sensillar ratios in *H. armigera* and *H. assulta*. Statistical significance was determined at the *P*<0.05 level.

Calcium imaging data were acquired by the software Till-vision (Till photonics) and analyzed by software ImageJ (NIH, USA) and custom-made programs in MATLAB (The Math Works, Inc). To compare responses of MGC-units to blends of different ratios, the paired *t*-test was used and statistical significance was determined at the *P*<0.01-level. To compare whole MGC responses to different blends, one-way ANOVA analysis was used and statistical significance was determined at the *P*<0.05-level.

In wind tunnel experiments, percentages of males performing sequential behaviors were subjected to Chi-square 2×2 test of independence with Yates’ continuity correction. The significance was determined at the *P*<0.05 level. All statistical analyses were carried out using SPSS software (release 16.0, SPSS Inc., Chicago, Illinois, USA).

## Supporting Information

Figure S1 **Physiological responses of two types of olfactory sensory neurons (OSNs) to different binary pheromone mixtures.** Ratios of the two compounds are Z11–16: Ald to Z9–16: Ald. **A**, typical neural records of the OSNs sensitive to Z11–16:Ald in *H. armigera* and to Z9–16:Ald in *H. assulta*. **B**, firing frequencies of the OSNs sensitive to Z11–16:Ald. **C**, firing frequencies of the OSNs sensitive to Z9–16:Ald. The tested dosage in different treatments was 10 µg. Paraffin oil was used as control. Stimulus duration was 500 ms. Values are mean ± SEM. The same letters above bars are not significantly different from one anther (ANOVA, *P*<0.05, *H. armigera*, n = 9; *H. assulta*, n = 15).
(TIF)

Figure S2 **The method to identify the activated area of Z9–16: Ald in the AL of** ***H. assulta*** **as an example.** A1: Spatial representation of 10 µg Z9–16: Ald in the AL of *H. assulta* by false-color coded images. A2: Gray scale image of AL in *H. assulta*. A3: Response activity of Z9–16: Ald (<50% hue) superimposed on grey scale images by ImageJ software with Z project treatment.
(TIF)
