# Supplementary figures and images for: Peripheral Coding of Sex Pheromone Blends with Reverse Ratios in Two Helicoverpa Species
Source: PLoS One. 2013 Jul 23;8(7):e70078. doi: 10.1371/journal.pone.0070078 (PMC3720945; doi:10.1371/journal.pone.0070078)

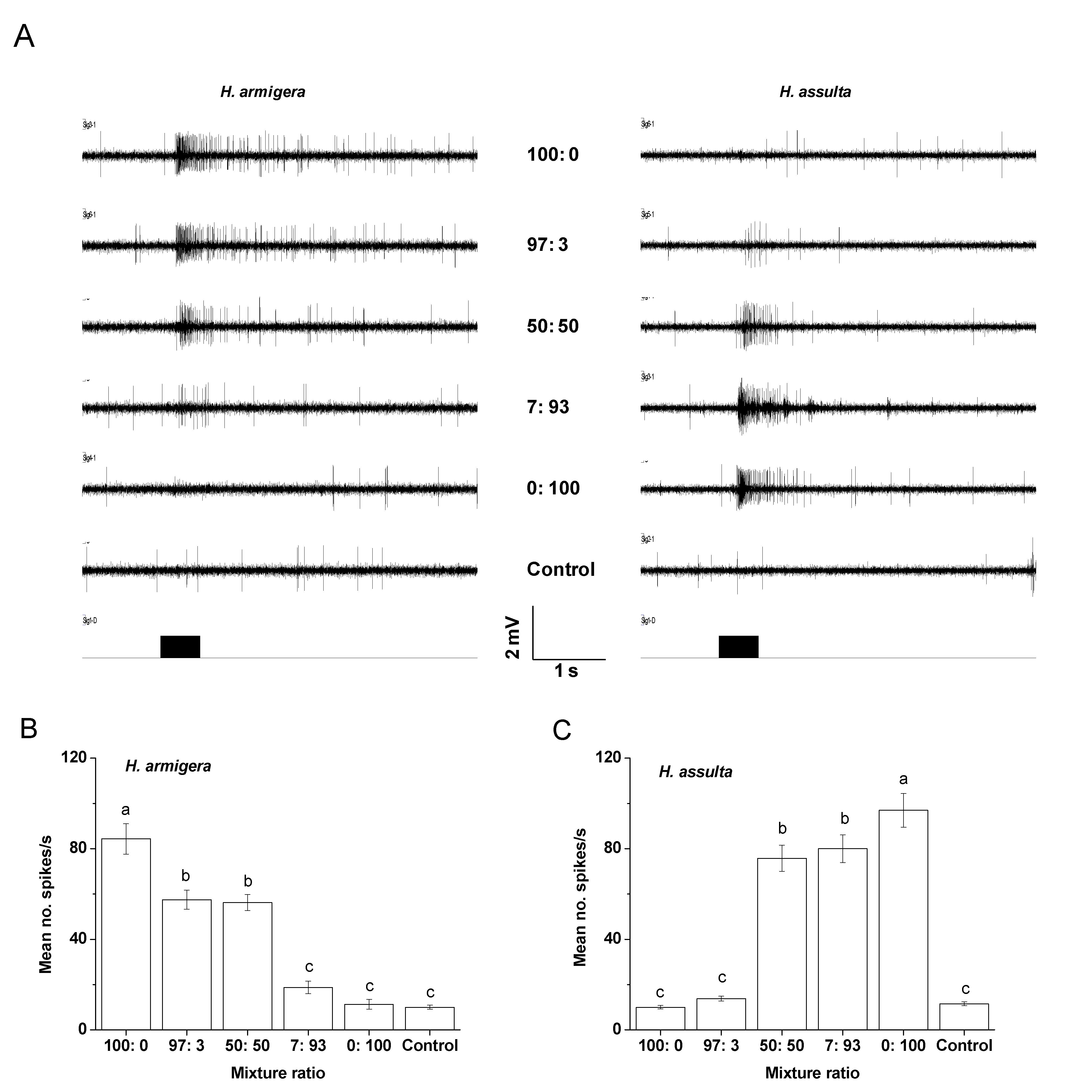

Supplement: Figure S1 — Physiological responses of two types of olfactory sensory neurons (OSNs) to different binary pheromone mixtures. Ratios of the two compounds are Z11–16: Ald to Z9–16: Ald. A, typical neural records of the OSNs sensitive to Z11–16:Ald in H. armigera and to Z9–16:Ald in H. assulta. B, firing frequencies of the OSNs sensitive to Z11–16:Ald. C, firing frequencies of the OSNs sensitive to Z9–16:Ald. The tested dosage in different treatments was 10 µg. Paraffin oil was used as control. Stimulus duration was 500 ms. Values are mean ± SEM. The same letters above bars are not significantly different from one anther (ANOVA, P<0.05, H. armigera, n = 9; H. assulta, n = 15). (TIF) [file pone.0070078.s001.tif]

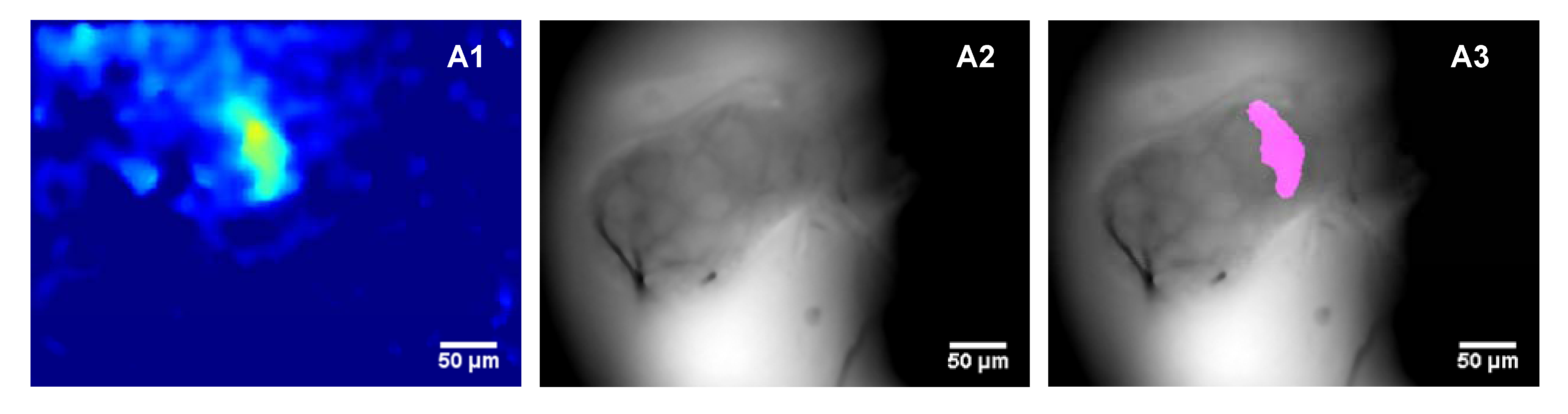

Supplement: Figure S2 — The method to identify the activated area of Z9–16: Ald in the AL of H. assulta as an example. A1: Spatial representation of 10 µg Z9–16: Ald in the AL of H. assulta by false-color coded images. A2: Gray scale image of AL in H. assulta. A3: Response activity of Z9–16: Ald (<50% hue) superimposed on grey scale images by ImageJ software with Z project treatment. (TIF) [file pone.0070078.s002.tif]
